# Correlation Between CASC8, SMAD7 Polymorphisms and the Susceptibility to Colorectal Cancer: An Updated Meta-Analysis Based on GWAS Results

**DOI:** 10.1097/MD.0000000000001884

**Published:** 2015-11-20

**Authors:** Kunhou Yao, Long Hua, Lunshou Wei, Jiming Meng, Junhong Hu

**Affiliations:** From the Department of General Surgery, Huaihe Hospital of Henan University, Kaifeng, Henan Province, China (KY, LH, JM, JH); and Department of Digestive Medicine, Huaihe Hospital of Henan University, Kaifeng, Henan Province, China (LW).

## Abstract

Genome-wide association studies (GWASs) and a number of case–control studies have suggested that several single nucleotide polymorphisms (SNPs), rs7837328, rs7014346, rs6983267, rs10505477 on CASC8 gene and rs4939827, rs4464148, rs12953717 on *SMAD7* gene are significantly correlated with the susceptibility to colorectal cancer (CRC). For the sake of clarifying the association, a meta-analysis was conducted and population heterogeneity was considered in the study.

A total of 34 articles including 90 studies (168,471 cases and 163,223 controls) that evaluated the relationship between the *CASC8*, *SMAD7* genes and the risk of CRC under the allelic model were reviewed. Also subgroup analysis was performed by ethnicity (Caucasian, Asian, and African) and all of the analyses were implemented in R 3.2.1 software.

Pooled data from the meta-analysis revealed that the A allele of rs7837328, the A allele of rs7014346, the G allele of rs6983267, the A allele of rs10505477, the T allele of rs4939827, the T of rs4464148, and the T of rs12953717 were significantly associated with an increased risk of CRC under the allelic model. Additionally, subgroup analyses of 6 SNPs by ethnicity (rs4464148 excepted) witnessed that the A allele of rs7837328, the G allele of rs6983267, and the T of rs12953717 were notably associated with an increased risk of CRC among Caucasian and Asian. Furthermore, the A allele of rs7014346, the A allele of rs10505477, and the T allele of rs4939827 were significantly related with an elevated risk of CRC only among Caucasian.

Our study suggested that for *CASC8* gene, SNP of rs7837328 and rs6983267 are risk factors for CRC among both Caucasian and Asian whereas rs7014346 and rs10505477 are risky gene polymorphisms only among Caucasian. For *SMAD7* gene, rs4939827 and rs4464148 are risk factors for CRC among Caucasian whereas rs12953717 could elevate the susceptibility to CRC in both Caucasian and Asian.

## INTRODUCTION

Colorectal cancer (CRC) is one of the most prevailing cancer occurred in the digestive system,^1^ and it ranked as the third primary cancer-causing death in the world.^2^ CRC is a multistep, multifactorial disease that results from various factors. Previous epidemiological studies have shown that lifestyle and dietary factors (smoking, unhealthy dietary intake, occupational exposures to chemicals, etc.) are common risk factors for the development of CRC.^3,4^ Although the pathogenesis of CRC is still unclear, molecular epidemiological studies have suggested that single nucleotide polymorphisms (SNPs) in genes play a vital role in CRC development and progression.^5,6^ Genome-wide association studies (GWASs) have revealed that genetic factors accounted for 33% of CRC cases in the world.^7–9^

Cancer susceptibility candidate 8 (*CASC8*) gene, a long noncoding RNA (lncRNA), is located in the region of 8q24.21, which is a nonprotein coding region.^10^ LncRNA is a new class of transcripts, which transcribed pervasively in the genome and regulates the expression of multiple genes.^11^ Studies have shown that SNPs in lncRNAs may affect the biological process of messenger RNA conformation, and result in the modification of its interacting partners.^12,13^ In addition to protein coding genes and microRNAs, the dysregulated expression of lncRNAs is likely to be pervasive in human cancers and can regulate tumorigenesis and predict tumor prognosis.^14^ Various cancer including prostate cancer,^15^ breast cancer, CRC, and gastric cancer have been reported to be correlated with the *CACS8* gene.^10^ Although some well-featured studies suggested the association between CASC8 gene SNP and the risk of CRC, few of them provided evidence that multiple SNPs in genes were correlated with the risk of CRC.

On the other hand, *SMAD7* gene, located in the region of 18q21, is one of the members of transforming growth factor-β (TGF-β) family signaling pathway. It has been proved to promote the antiinflammatory effects of TGF-β signaling via binding to TAB2 and TAB3 and inhibiting TAK1.^16,17^ Apart from that, TGF-β plays an important role in promoting metastasis in many solid tumors.^18^ Studies have indicated that *SMAD7* gene is related with breast cancer,^19^ gastric cancer,^20^ pancreatic cancer,^21^ CRC,^22^ and so on. Great efforts have been made to investigate the association between *SMAD7* gene polymorphisms and the risk of CRC. Nevertheless, the functional significance of these SNPs is still unclear.

Several studies have evaluated the association between parts of gene polymorphisms and the susceptibility to CRC risk, but the associations suggested by different studies were inconsistent. Besides that, a single case–control study may fail to discover the effects of gene polymorphisms on the susceptibility to CRC due to various genotypes and the small sample size. However, meta-analysis has the advantage of increased statistical power and reduced random error which could generate more accurate statistical results than that in individual studies. As a result of this, a meta-analysis with eligible studies was carried out to provide an integrated understanding of the impact of *CACS8* and *SMAD7* gene polymorphisms on the susceptibility to CRC. Subgroup analyses were performed in order to explore potential sources of heterogeneity among individual studies.

## METHODS

Ethical approval was not necessary for the current meta-analysis.

### Search Strategy and Selection

A meta-analysis was carried out based on the guidelines of PRISMA (Preferred Reporting Items for Systematic Reviews and Meta-Analyses) statement.^23^ Using the following MeSH searching terms: “colorectal cancer,” “polymorphism,” “rs7837328,” “rs7014346,” “rs6983267,” “rs10505477,” “rs4939827,” “rs4464148,” “rs12953717,” “case–control,” and “meta-analysis,” articles were searched electronically in PubMed, MEDLINE, and Embase without language restrictions. Additionally, the reference lists in each relevant article were searched manually for other related publications.

### Data Extraction

The following criteria were used as the inclusion criteria for relevant studies: patients in the study were diagnosed with CRC at any tumorigenesis stage; subjects in the control group were healthy and free from cancer or neoplasm; availability of genotype or allele of the case and control groups or minor allele frequency (MAF) of the case and control group or related odds ratio (OR) and confidence interval (95% CI) for the allelic model of CRC; and genotype distributions complied with Hardy–Weinberg equilibrium. The following criteria were set as the primary exclusion criteria: CRC patients with other cancer; no available data of genotype or allele frequencies or MAF or related OR; abstracts and reviews; and duplicated publications.

These studies were screened by 2 independent investigators and relevant information was individually extracted from all qualified publications. Disagreements between the 2 investigators were recorded and settled by a discussion with a third investigator. Finally, the following information was collected from each qualified study: author of surname, year of publication, ethnicity of subjects, sample sizes of the case and control group, frequency distributions of genotype and allele, OR and 95% CI of the allelic model.

### Statistical Analysis

A Chi-squared (χ^2^) test was conducted to investigate the heterogeneity among individual studies. The fixed-effects model was used if there was no significant heterogeneity among individual studies (I^2^ < 50%, *P* > 0.05), otherwise, the random-effects model was applied in the meta-analysis. Pooled ORs were calculated for the allelic model to estimate the association between *CASC8*, *SMAD7* gene polymorphisms and CRC. Moreover, Z test was used to assess the statistical significance of the pooled OR and Begg funnel plot was carried out to determine whether or not there was significant publication bias. A value of *P* < 0.05 suggests that statistically significant bias was presented in the meta-analysis. All of the statistical analyses were implemented using R 3.2.1 software and a 2-tailed *P*-value of less than 0.05 was considered as the significant level.

## RESULTS

### Study Characteristics

**FIGURE 1:**
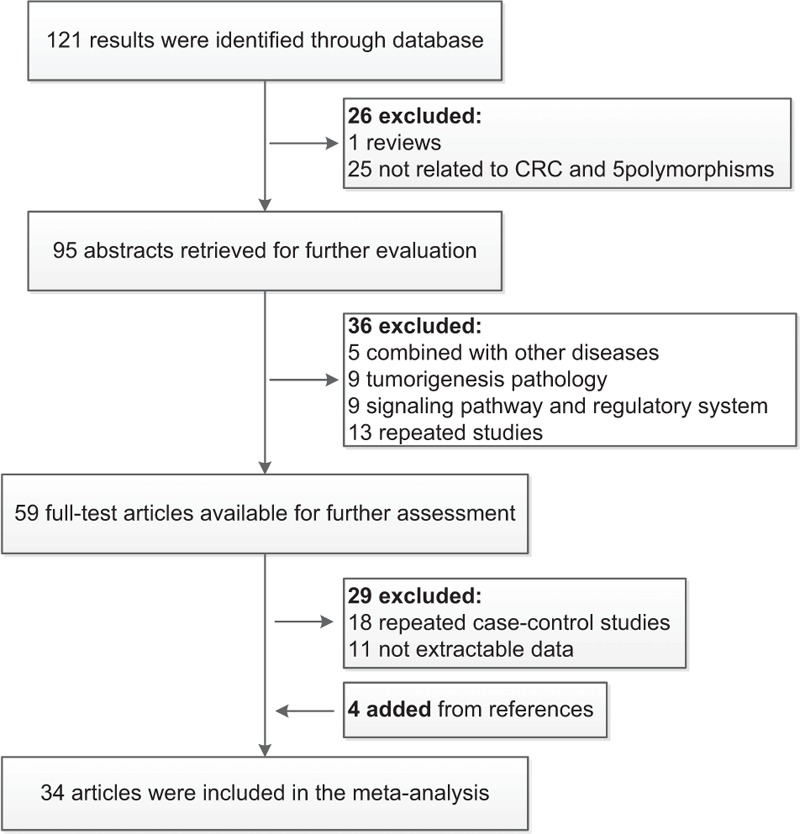
Studies selection flowchart the meta-analysis.

As shown in Figure 1, 121 reports were selected initially using the MeSH searching terms and 91 articles were excluded after initial screening of titles and abstracts. Eventually, 34 articles met the inclusion criteria and 4 extra reference articles were added using manual searching. Newcastle-Ottawa Scale (NOS) was used for quality assessment, and all of the 34 studies achieved moderately high quality with scores above 6 (Supplement Figure S1). The main characteristics of included studies were shown in Supplement Table S1. There were 34 articles including 90 case–control studies in total and some of the articles contained multiple studies as they investigated multiple SNPs and different ethnicity. Furthermore, a total of 90 studies were further classified by ethnicity (Asian, African, and Caucasian) for subgroup analysis. Among included studies, 4 studies were conducted on rs7837328 (6167 cases and 5978 controls), 13 studies on rs7014346 (22,685 cases and 20,794 controls), 27 studies on rs6983267 (47,461 cases and 46,958 controls), 11 studies on rs10505477 (15,584 cases and 17,613 controls), 21 studies on rs4939827 (47,029 cases and 43,779 controls), 4 studies on rs4464148 (12,508 cases and 11,337 controls), and 10 studies on rs12953717 (17,037 cases and 16,764 controls).

### Meta-Analysis Results

**FIGURE 2:**
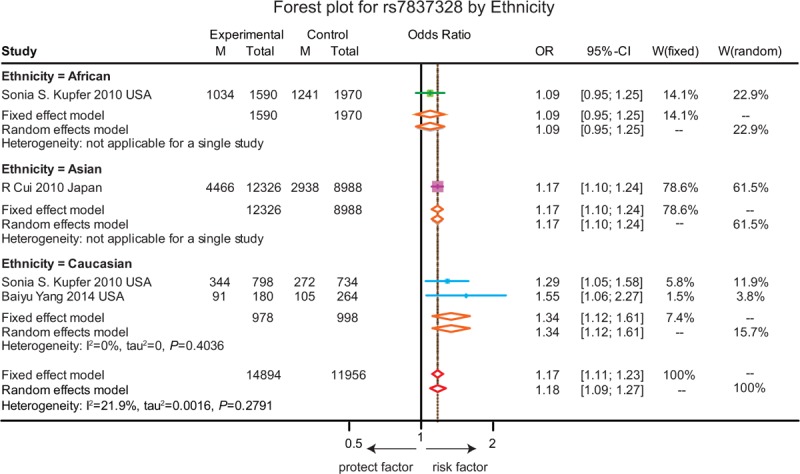
Forest plot for gene polymorphism of rs7837328 by ethnicity.

**FIGURE 3:**
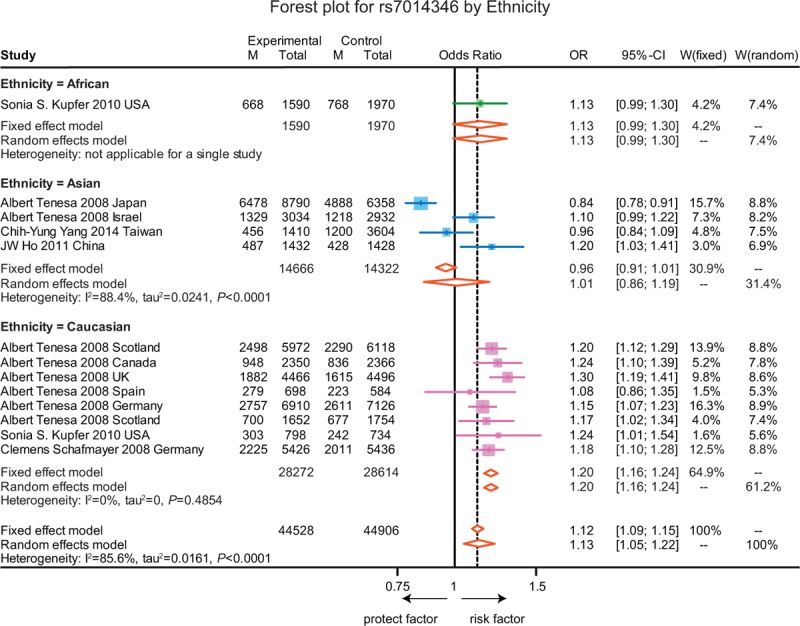
Forest plot for gene polymorphism of rs7014346 by ethnicity.

**FIGURE 4:**
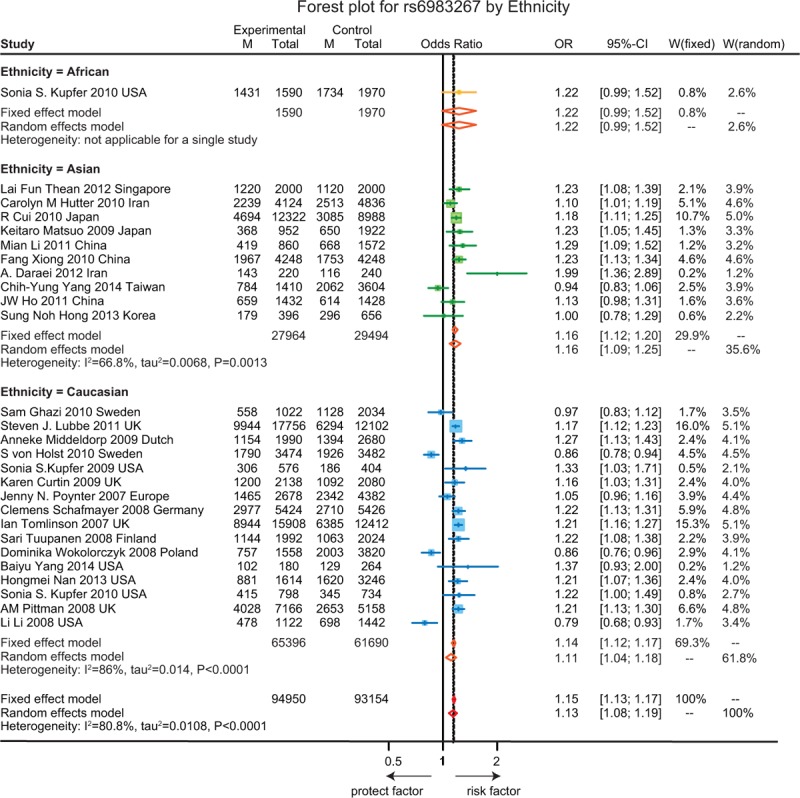
Forest plot for gene polymorphism of rs6983267 by ethnicity.

**FIGURE 5:**
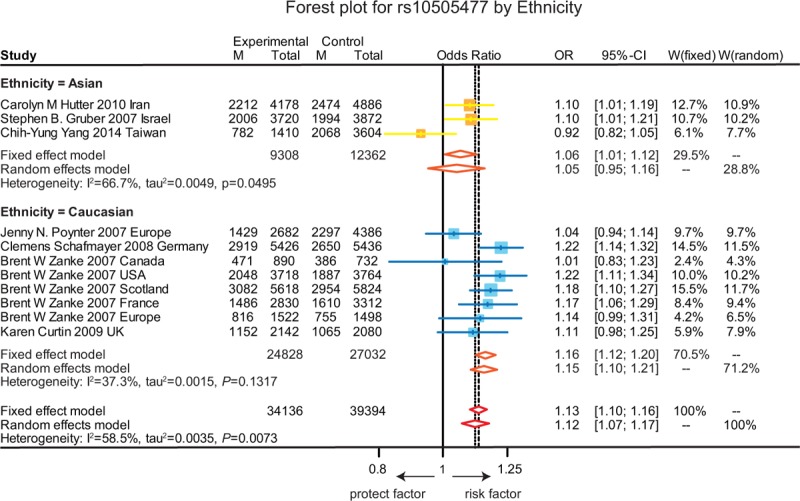
Forest plot for gene polymorphism of rs10505477 by ethnicity.

**FIGURE 6:**
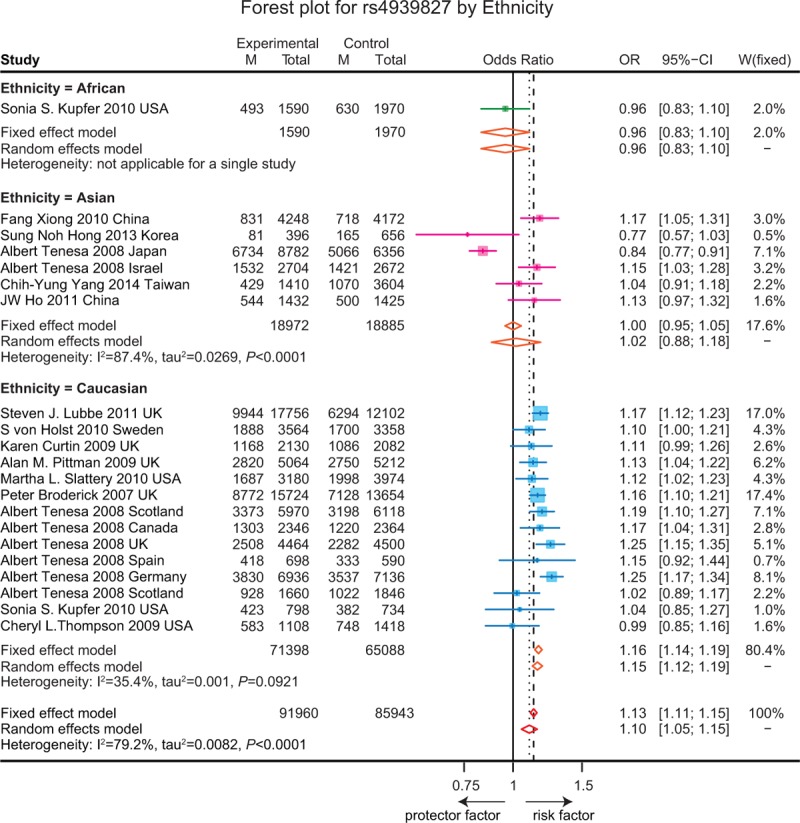
Forest plot for gene polymorphism of rs4939827 by ethnicity.

**FIGURE 7:**
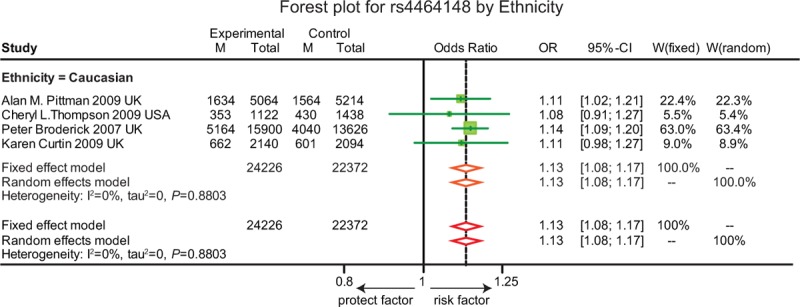
Forest plot for gene polymorphism of rs4464148 by ethnicity.

**FIGURE 8:**
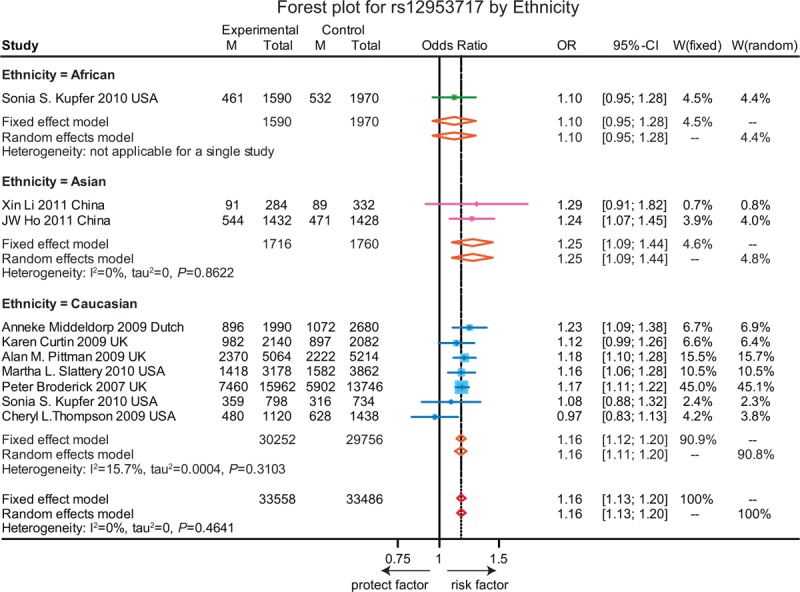
Forest plot for gene polymorphism of rs12953717 by ethnicity.

The association between the above 7 SNPs on gene *CASC8*, *SMAD7* and with the susceptibility to CRC in the allelic model are revealed in Table 1. A detailed analysis about the relationship between each specific SNP and the susceptibility to CRC is presented in Figures 2–8.

**TABLE 1:**
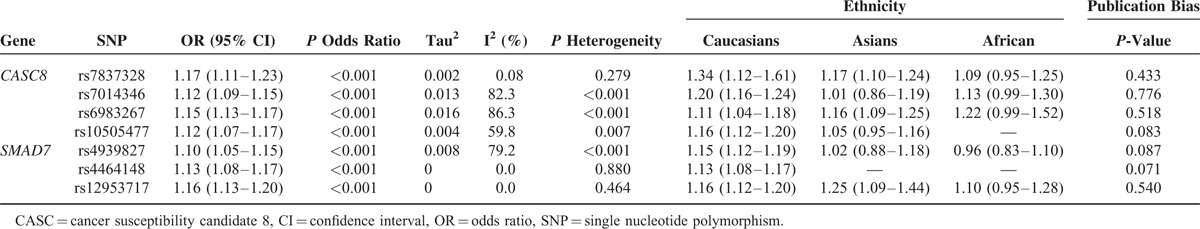
Meta-Analysis of 7 Polymorphisms in CASC8 Gene and SMAD7 Gene and Colorectal Cancer Susceptibility

### Association Between *CASC8* Gene Polymorphisms and CRC Risk

Four SNPs on *CASC8* gene were analyzed in the current meta-analysis. Studies on rs7837328 showed a low heterogeneity (Tau^2^ = 0.002, I^2^ = 0.08%, *P* = 0.279) and therefore a fixed-effects model was conducted. On the other hand, studies on the other three SNPs (rs7014346, rs6983267, rs10505477) indicated significant heterogeneity (Tau^2^ = 0.013, I^2^ = 82.3%, *P* < 0.001; Tau^2^ = 0.016, I^2^ = 86.3%, *P* < 0.001; Tau^2^ = 0.004, I^2^ = 59.8%, *P* = 0.007, respectively) and hence a random-effects model was used in the meta-analysis.

As shown in Figure 2, the A allele of rs7837328 was significantly associated with an increased risk of CRC as compared to the G allele (OR = 1.17, 95% CI = 1.11–1.23, *P* < 0.001). In addition, subgroup analyses by ethnicity revealed that the A allele of rs7837328 was evidently related with an increased risk of CRC in Caucasian and Asian (OR = 1.34, 95% CI = 1.12–1.61; OR = 1.17, 95% CI = 1.10–1.24). However, this association was not significant in the African group (OR = 1.09, 95% CI = 0.95–1.25). Similarly, the A allele of rs7014346 was significantly associated with an increased risk of CRC (OR = 1.12, 95% CI = 1.09–1.15, *P* < 0.001). However, this correlation was only significant in the Caucasian group and there is no such correlation in the Asian and African groups (OR = 1.20, 95% CI = 1.16–1.24; OR = 1.01, 95% CI = 0.86–1.19; OR = 1.13, 95% CI = 0.99–1.30) (Figure 3). Furthermore, the results suggested that the G allele of rs6983267 was significantly associated with an increased risk of CRC (OR = 1.15, 95% CI = 1.13–1.17, *P* < 0.001) and a similar correlation was presented in the A allele of rs10505477 (OR = 1.12, 95% CI = 1.07–1.17, *P* < 0.001). For rs6983267, subgroup analyses indicated that there the G allele was significantly associated with the susceptibility to CRC among Caucasian and Asian (OR = 1.11, 95% CI = 1.04–1.18; OR = 1.16, 95% CI = 1.09–1.25), while such correlation was not significant in the African group (OR = 1.22, 95% CI = 0.99–1.52) (Figure 4). For rs10505477, subgroup analyses of Caucasian and Asian were carried out due to the lack of African studies and it revealed that the A of rs10505477 was significantly related with an increased susceptibility to CRC in the Caucasian group (OR = 1.16, 95% CI = 1.12–1.20), while no such significant association was observed in the Asian group (OR = 1.05, 95% CI = 0.95–1.16) (Figure 5).

### Association Between SMAD7 Genetic Polymorphisms and CRC Risk

For *SMAD7* gene, 3 polymorphisms (rs4939827, rs4464148, rs12953717) were analyzed in this study and significant associations between these SNPs and the susceptibility to CRC were indicated by our analysis. A random-effect model was applied to the SNP of rs4939827 as significant heterogeneity was presented in individual studies (Tau^2^ = 0.008, I^2^ = 79.2%, *P* < 0.001). Conversely, fixed-effects models were applied to SNPs of rs4464148 and rs12953717 as no significant heterogeneity was presented in these 2 SNPs in individual studies (Tau^2^ = 0, I^2^ = 0.0%, *P* = 0.880; Tau^2^ = 0, I^2^ = 0.0%, *P* = 0.464).

As revealed in Table 1, the T allele of rs4939827 was significantly related with an increase risk of CRC (OR = 1.10, 95% CI = 1.05–1.15, *P* < 0.001). Subgroup analysis based on ethnicity indicated a similar correlation between SNP in rs4939827 and the susceptibility to CRC in the Caucasian group whereas such a correlation was not presented in the Asian and African group (Figure 6). Furthermore, the T allele of rs4464148 and the T allele of rs12953717 were significantly associated with an elevated risk of CRC (OR = 1.13, 95% CI = 1.08–1.17, *P* < 0.001; OR = 1.16, 95% CI = 1.13–1.20, *P* < 0.001). However, the subgroup analysis of rs4464148 was not applicable as no studies were found in the Asian and African populations (Figure 7) Furthermore, the T allele of rs12953717 was significantly associated with an increased the risk of CRC among Caucasian and Asian (OR = 1.16, 95% CI = 1.12–1.20; OR = 1.25, 95% CI = 1.09–1.44), while such an association was not presented in the African group (OR = 1.10, 95% CI = 0.95–1.28) (Figure 8).

### Publication Bias

As suggested in Table 1 and Supplement Figure S2a–g, no significant asymmetry could be observed in the funnel plots and results from Begg funnel plot suggested that there was no significant publication bias presented in the study (*P* = 0.433 for rs7837328, *P* = 0.776 for rs7014346, *P* = 0.518 for rs6983267, *P* = 0.083 for rs10505477, *P* = 0.087 for rs4939827, *P* = 0.071 for rs4464148, and *P* = 0.540 for rs12953717).

## DISCUSSION

Recently, a lot of attention has been paid to gene polymorphisms involved in tumorigenesis due to the fast growing interests in cancer research. GWASs have contributed substantially to the identification of common genetic variants related to human cancer. Several studies evidenced that genetics play a critical role in CRC development and progression,^24–26^ and GWASs have suggested that various genes were associated with the susceptibility to CRC.^27^ Moreover, recent evidence suggested that *CASC8* and *SMAD7* gene polymorphisms both play important roles in different cancers including prostate cancer^28,29^ and breast cancer.^19,30^ Furthermore, there seems to exist strong associations between gene polymorphisms in *CASC8* (rs7837328, rs7014346, rs6983267, rs10505477), *SMAD7* (rs4939827, rs4464148, rs12953717), and an increased risk of CRC.^8,17,31–37^ However, the correlation between these gene polymorphisms and the susceptibility to CRC is still unclear due to various conclusions drawn by individual studies.

For the purpose of providing a comprehensive and consistent conclusion, a meta-analysis of 90 independent case–control studies was carried out and how SNP rs10505477, rs7837328, rs7014346, and rs6983267 located on *CASC8* in the 8q24 region and SNP rs4939827, rs4464148, rs12953717 in the *SMAD7* region affect CRC tumorigenesis was investigated. Researchers have shown that *CASC8* and *SMAD7* genes are highly correlated with CRC development and progression.^38–40^ Subgroup analyses by ethnicity indicated that SNPs of rs7014346, rs10505477, and rs4939827 were significantly associated with an increased risk of CRC in the Caucasian group, whereas SNPs of rs7837328, rs6983267, and rs12953717 were considered as significant risk factors for CRC in both of the Caucasian and Asian group. Besides that, rs4464148 is a risk factor for CRC development and progression in the Caucasian group and the association between rs4464148 SNP and the susceptibility to CRC was unknown in the Asian and African groups due to the lack of relevant studies.

Despite the fact that well-established biological pathways contribute to the majority of CRC risk variants, the functions of some reported loci are still abstruse. The association between multiple SNPs in the chromosome region 8q24 and the increased risk of several solid tumor malignancies, including CRC, has been reported by various independent studies.^10,31,41,42^ Recent GWASs have suggested that SNP of rs7837328 is significantly correlated with the risk of CRC.^43–45^ As a result of this, it has been hypothesized that rs7837328 may function through its long-range linkage with causal variants contained in other oncogenes or tumor suppressor genes. Additionally, others have speculated that SNP of rs7837328 may influence the gene expression through long-range *cis*-regulatory elements.^46^ The association between rs6983267 and CRC was firstly proposed in 2007 by 3 GWAS,^43,45,47^ and further investigated by several case–control studies. Tuupanen et al^48^ reported that rs6983267 might enhance Wnt signaling via affecting binding to T-cell factor-4 (TCF4). Apart from that, the G allele of rs6983267 has been conferred to increase the risk of CRC by interacting with the promoter of MYC oncogenes,^48–50^ which was an aberrant expression in numerous tumors, including gastric cancer.^51^ Moreover, a relationship between SNP of rs7014346 and CRC susceptibility was discovered in a GWAS^44^ and previous study has also suggested that the GA genotype of rs7014346 was significantly associated with a decreased risk of breast cancer. The rs10505477 SNPs, also located in the chr.8q24 region, were significantly associated with the risk of CRC^43,45,52^ and breast cancer.^53,54^ Ma et al^10^ hypothesized that rs10505477 in LncRNA *CASC8* was involved in gastric cancer progression and it might serve as a potential prognosis marker in the Chinese population.

GWAS have identified several genomic regions associated with the risk of CRC and these genomic regions include genes in the TGF-β signaling pathway such as *SMAD7*,^44,55^*BMP2*,^26,33,56^*BMP4*,^26,56^ and *GREM1*.^26,57^ Phipps et al^58^ found that rs4939827, located in *SMAD7* intron 4, was significantly associated with poorer overall survival status of patients and disease-specific survival status. Rs4939827, encoding an intracellular antagonist of the TGF-β pathway frequently, was revealed to be inactivated in CRC^44,55,59–61^ and it explains approximately 1% of the familial relative risk of CRC in East Asian.^22^ Another study revealed the association between SNP of rs4939827 and the risk of CRC in the Croatian population.^36^ Rs4464148, located in intron 3 of *SMAD7* gene, has also been discovered to be correlated with the risk of CRC in European-ancestry populations.^55^ Besides that, Rs4464148 variant genotype of *SMAD7* has been proved to be associated with an increased cancer incidence.^62^ Meanwhile, rs4464148 has been used for delimiting new groups with high-risk CRC in clinical practice, especially in Poland, Estonia, and Lithuania.^63^ Dai et al^64^ discovered that the homozygous variant genotype of rs4464148 was significantly associated with better survival status in CRC stage III patients, as compared with patients having the homozygous wild-type and heterozygous genotypes. Additionally, the rs12953717 of *SMAD7*, which was reported in 2 GWAS studies,^44,55^ may be valuable markers for predicting the risk of tumor formation. Empirical evidence suggested that rs12953717 was a common risk marker of lung, colorectal, and gastric cancer in the Chinese Han population.^65^ Li et al^65^ discovered that rs12953717 confers a strong association with the above 3 cancer in the Chinese Han population. More importantly, rs12953717 has been discovered to be significantly associated with an increased risk of CRC.^17,66^

Subgroup analyses revealed that rs7837328, rs12953717, and rs6983267 polymorphisms were significantly associated with an increased risk of CRC in both Asian and Caucasian, while rs7014346, rs4939827, and rs10505477 polymorphisms were significantly associated with an increased risk of CRC in Caucasian. Due to the lack of studies among Asian and African, subgroup analyses were not implemented for rs4464148 which was found to be significantly associated with an increased risk of CRC in Caucasian. The difference in the association among different ethnicities may result from other factors such as socioeconomic environment and race.

Although some inconsistent conclusions on the association between SNPs and the susceptibility to CRC have been clarified in the meta-analysis, some limitations of the meta-analysis should be taken into account. First of all, the limited amount of data did not enable us to determine how the interaction between various gene polymorphisms affects the susceptibility to CRC. Similarly, potential interactions between genetic and nongenetic factors could not be evaluated due to the lack of data. As a result of this, results from the meta-analysis might be affected by various confounding factors and effect modification might be presented in the statistical analysis. To the best of our knowledge, this is the most comprehensive meta-analysis which individually examined the correlation between seven gene polymorphisms and the susceptibility to CRC. Overall, the meta-analysis revealed that all of the 7 SNPs (rs7837328, rs7014346, rs6983267, rs10505477, rs4939827, rs4464148, rs12953717) were significantly associated with the risk of CRC. As a result of this, all of the 7 gene polymorphisms could be considered as potential risk markers for detecting and diagnosing CRC and it may play an important role in the early prophylaxis and control measures to reduce the incidence of CRC. However, race and ethnicity might have significant impact on the detection and diagnose using gene polymorphisms as subgroup analysis indicated that the association between the 7 SNP and the risk of CRC varied significantly in different ethnicities. It is significantly recommended that well-design studies which incorporate different ethnicities together with genetic and nongenetic factors should be carried out in order to assess the interaction between different gene polymorphisms which might be considered as the next benchmark for detecting CRC.
